# Precision mentoring (PM): a proposed framework for increasing research capacity in health-related disciplines

**DOI:** 10.1080/10872981.2021.1964933

**Published:** 2021-08-24

**Authors:** Lynda B. Ransdell, Heidi A. Wayment, Anna L. Schwartz, Taylor S. Lane, Julie A. Baldwin

**Affiliations:** a Southwest Health Equity Research Collaborative (SHERC) in the Center for Health Equity Research (CHER), Northern Arizona University, Flagstaff, AZ, USA; b College of Health and Human Sciences, Northern Illinois University, DeKalb, IL, USA; c Department of Psychological Sciences, Northern Arizona University, Flagstaff, AZ, USA; d School of Nursing, Northern Arizona University, Flagstaff, AZ, USA; e Department of Health Sciences, Northern Arizona University, Flagstaff, AZ, USA

**Keywords:** Faculty development, research success, faculty of color, new faculty, diversity

## Abstract

**Problem:**

Research productivity is expected of academic faculty, and mentoring can facilitate it. This paper presents a framework for using mentoring to develop researchers in health disciplines.

**Approach:**

We utilized recent literature reviews, and experience developing researchers at an emerging research institution within the Research Centers for Minority Institutions (RCMI) program, to propose a precision mentoring (PM) framework for research development.

**Outcomes:**

Although we cannot precisely determine *how much* improvement was due to the PM framework, over the 4 years of our program, the quality and quantity of pilot project proposals (PPP) has increased, the number of external proposals submitted and funded by PPP investigators has increased, and the number of faculty participating in our program has increased. Surveys distributed to our 2021–22 PPP applicants who did not receive funding (n = 5/6 or 86.7%) revealed that new investigators most frequently sought mentoring related to career guidance (e.g., institutional culture, pre-tenure survival strategies), grant proposal basics (e.g., working with funding agencies, reviewing aims, balancing priorities, and enhancing scientific rigor), and identifying funding opportunities.

**Next Steps:**

We recommend shifting the mentoring paradigm such that: (a) mentees are pre-screened and re-screened for their current skill set and desired areas of growth; (b) mentoring occurs in teams vs. by individuals; (c) mentors are trained and rewarded, and (d) attention is paid to enhancing institutional culture.

## Problem

Research productivity, (e.g., peer-reviewed publications and external grants), is one of the most important components of success in higher education. Mentoring is an essential component for helping faculty succeed in higher education, and numerous literature reviews and meta-analyses have espoused the benefits of mentoring [1–6]. Although most existing research on mentoring has focused on mentoring new and early-stage faculty, it is also important to specifically consider the needs of underrepresented minority (URM) faculty (e.g., racial and ethnic minorities), women in some disciplines, and mid-career faculty [1]. Given the importance of mentoring, the purpose of this paper is to describe a framework for precision mentoring (PM).

## Outcomes

In a recent literature review covering the 10 years of mentoring research, we examined personal, interpersonal, and institutional barriers and facilitators related to research productivity in new investigators (NI), early-stage investigators (ESI) and underrepresented minority faculty (UMF), also known as underrepresented minorities (URM) [1]. For definitions of these terms, see https://grants.nih.gov/grants/esi-status.pdf. Mid-career faculty were not included in our literature review, but they are important to include.

### Pre-assessing and re-assessing mentoring needs

Table 1 contains a checklist, administered as a questionnaire followed by an interview, for identifying research barriers and facilitators for mentees. The strategy of assessing baseline research readiness and re-evaluating regularly is well-supported **[**7–12**]**.

**Table 1.** Topics used to assess research barriers and facilitators for NI, ESI and UMF. Below is a list of topics we have used to guide discussions about factors related to research productivity in pre- and re-assessment of NI, ESI and UMF

Barriers/Facilitators
**PERSONAL**
Personal Accountability
Time management skills/experiences
Work/life balance issues
Sense of connection/isolation in university/unit
Experiences of bias and discrimination
Power dynamics in unit/university/mentoring
Career planning skills
Leadership Skills
Resilience, grit and personal resources
**INTERPERSONAL**
Strategies for expanding professional network/research collaborators
Advocacy for diversity and cultural humility within university/unit
Negotiation skills in team research settings
Managing a research team (peers, students, trainees, research coordinators etc.)
**TECHNICAL**
Manuscript writing skills
Grant writing skills
Statistical analysis skills
Designing and delivering professional presentations
Marketing and graphic design skills
**INSTITUTIONAL**
Access to Mentorship
Access to content, synthesis, and analytical expertise (research skill development)
Mentorship training & effectiveness
Research training and skill development
Research resources (lab space, graduate students, post-docs, lab technicians)
Support for professional activities (conference travel, networking)
Support for science culture (i.e., research in unit/university)
Teaching load reduction or buyout opportunities
Service load (Underrepresented Minority Faculty tax)
Understanding of promotion/tenure procedures
Succession planning after completion of mentoring program
Pre/post award support for sponsored projects
Culturally responsive mentoring and cultural humility emphasized within institution
Culturally responsive research training and education

### Research plan development

After needs are assessed, the mentee and mentors develop a research plan for the mentee that includes outcome and process goals, and potential individuals and areas of mentor/mentee collaboration. Table 2 presents a sample research plan for an investigator. Specifically, in this example, the mentee and mentor decide upon 4 outcome goals related to research success (e.g., publish, write a grant, manage time, work toward tenure). Each outcome goal has process goals, or strategies for achieving desired outcome or overall goals. To accomplish these goals, additional mentors are recruited, and their responsibilities are denoted with an ‘X’ placed in the month or months that mentor and mentee should meet.

**Table 2.** Sample research plan for NI, ESI or UMF

Research Mentoring Activities	Mentor 1				Mentor 2				Mentor 3				Mentor 4				Mentor 5			
**OVERALL GOAL 1: Publish a paper in area of expertise (may or may not separate multiple papers – depending on topic)**	August	November	February	May	August	November	February	May	August	November	February	May	August	November	February	May	August	November	February	May
IRB submission/approval	X			X		X	X													
Recruit subjects		X	X		X			X										X	X	X
Community Connections		X			X			X										X	X	X
Mixed Methods Analysis knowledge	X		X																	
Collaborator and Network Builder	X	X					X	X					X			X				
Knowledge of Manuscript Submission and Re-submission Process			X	X									X	X						
**OVERALL GOAL 2: Submit NIH R01**																				
Knowledge of Office of Sponsored Projects	X	X					X	X												
Knowledge of and Experience with NIH funding		X	X	X	X															
Content Knowledge	X		X			X		X	X	X			X	X	X	X				
**OVERALL GOAL 3: Manage time better** **and find more work/life balance**																				
Also has caregiving responsibilities									X	X	X	X	X	X	X	X				
Same ethnic or gender background													X	X	X	X				
Time Management & Goals									X	X	X	X					X			
Mentoring expert									X	X	X	X					X			
**OVERALL GOAL 4: Track & Evaluate Tenure Progress**																				
Keep professional activity records up-to-date (annual evaluation/tenure review process)	X	X	X	X					X											
Work to Lessen Implicit Bias	X					X			X	X	X	X			X					X

Process goals are listed below OVERALL GOALS.

### Mentoring team assignments

As soon as mentoring needs are identified and a plan is developed, a team of mentors is recruited. In addition to identifying members of mentoring teams, mentors should be trained in ‘best practices of mentoring’ and regularly evaluated. Readers are referred to the National Research Mentoring Network website (see: https://nrmnet.net/mentorship-training-programs/) **[**13,14**]**, for best practices for training research mentors.

### Sample PM framework for grant writing

**Figure 1.**
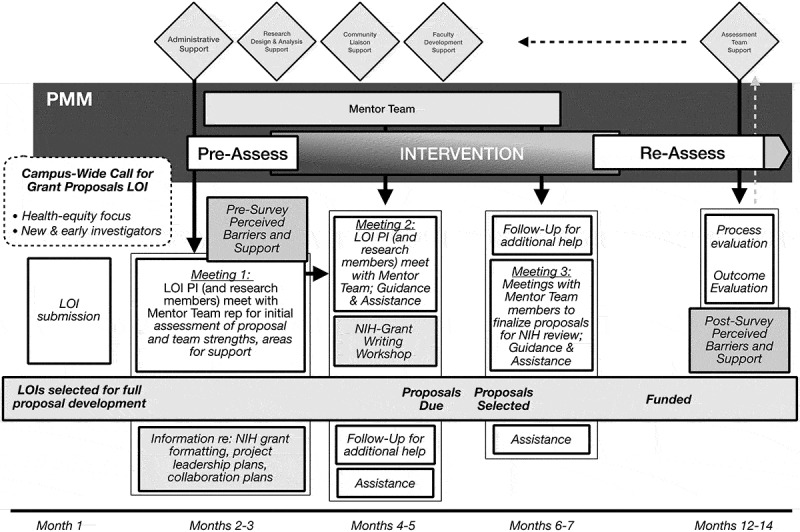
Applying the precision mentoring framework to grant proposal development

Figure 1 illustrates how our Precision Mentoring Framework is applied toward grant writing at our university. At the beginning of each academic year (See Month 1 in Figure 1), we issue a campus-wide call for pilot project proposals (PPPs), focused on biomedical, behavioral, and clinical research that improves health equity or decreases health disparities. Faculty submit a letter of intent (LOI) that outlines the importance of their research, specific aims, how they will develop and assess these aims, and how their project contributes to health equity. Individuals interested in submitting a LOI can attend an informational workshop, designed to discuss ideas and provide ‘best practices’ for submissions. Investigators then submit LOIs, and the strongest LOIs are invited to submit full proposals. The full proposal format includes instructions and scoring criteria used by the National Institutes of Health (NIH), and it requires a summary of project leadership and collaborations.

During months 2 and 3, those who are selected to submit full proposals have a meeting with senior faculty for an assessment of strengths and weaknesses of their proposal. Over the course of the next few months, a second meeting is scheduled with mentor teams who have content knowledge, statistical/methodological expertise, and community connections. An NIH grant writing workshop is presented to help investigators put their proposals into final format.

After pilot proposals are selected, monthly meetings are held with the mentee and mentoring team to assess progress, make recommendations, and solve problems. Investigators with funded pilot projects are encouraged to submit full NIH proposals, and publish papers related to their grants. Re-assessment of mentoring goals, strategies, successes and failures occurs regularly, and re-assignment of mentors occurs when necessary. Individuals who do not receive pilot funding are queried about the mentoring they received, and they are encouraged to continue with mentoring.

## Next steps

Researchers should continue to test the PM framework. It is important to determine whether regularly screening and individualizing the mentoring approach, having a team of mentors, training mentors, and being attentive to campus research culture will result in more successful research development. We should also study the impact of mentoring within specific disciplines and varying faculty ranks. We know that mentoring is beneficial, yet we need to go further to answer ‘how’ mentoring works. Developing additional scales to measure mediators and moderators of mentoring success would help us determine which facilitators are most important, specific to discipline, institution, and resources.
